# Evolution of bacteria specialization along an antibiotic dose gradient

**DOI:** 10.1002/evl3.52

**Published:** 2018-05-08

**Authors:** Noémie Harmand, Romain Gallet, Guillaume Martin, Thomas Lenormand

**Affiliations:** ^1^ CEFE, CNRS, Univ Montpellier Univ Paul Valéry Montpellier 3, EPHE, IRD Montpellier France; ^2^ UMR BGPI, INRA, Montpellier SupAgro Univ. Montpellier, Cirad, TA A‐54/K Montpellier Cedex 5 France; ^3^ Institut des Sciences de l'Evolution de Montpellier UMR CNRS‐UM II 5554, Université Montpellier II 34 095 Montpellier cedex 5 France

**Keywords:** *Escherichia coli*, experimental evolution, fitness landscape, specialization

## Abstract

Antibiotic and pesticide resistance of pathogens are major and pressing worldwide issues. Resistance evolution is often considered in simplified ecological contexts: treated versus nontreated environments. In contrast, antibiotic usually present important dose gradients: from ecosystems to hospitals to polluted soils, in treated patients across tissues. However, we do not know whether adaptation to low or high doses involves different phenotypic traits, and whether these traits trade‐off with each other. In this study, we investigated the occurrence of such fitness trade‐offs along a dose gradient by evolving experimentally resistant lines of *Escherichia coli* at different antibiotic concentrations for ∼400 generations. Our results reveal fast evolution toward specialization following the first mutational step toward resistance, along with pervasive trade‐offs among different evolution doses. We found clear and regular fitness patterns of specialization, which converged rapidly from different initial starting points. These findings are consistent with a simple fitness peak shift model as described by the classical evolutionary ecology theory of adaptation across environmental gradients. We also found that the fitness costs of resistance tend to be compensated through time at low doses whereas they increase through time at higher doses. This cost evolution follows a linear trend with the log‐dose of antibiotic along the gradient. These results suggest a general explanation for the variability of the fitness costs of resistance and their evolution. Overall, these findings call for more realistic models of resistance management incorporating dose‐specialization.

The massive increase in use of antibiotics since 1950 has greatly improved standards of living in our societies but has led to an explosion in the number of resistant phenotypes in microorganisms (World Health Organization 2014). Besides being naturally excreted by microorganisms, antibiotics are also massively used for medical, agronomical, veterinary, and industrial purposes. Antibiotics occur in concentration gradients (hereafter doses). Within the body of treated animals or humans, the antibiotic molecules are differentially absorbed, distributed, and eliminated, resulting in large variations of doses (Levison and Levison 2009). These gradients also occur in the environment as antibiotic molecules easily diffuse over long distances and pollute soils and water (Thiele‐Bruhn 2003; Kümmerer 2009; Depledge 2011). Thus, from hospitals to farms to rivers to soils, a very large panel of antibiotic molecules is present at varying doses.

A central tenet of evolutionary ecology is that different ecological conditions select for distinct adaptations, that is diverging phenotypes. This view applies to gradual ecological changes. For instance, the beak size and shape of Darwin finches evolved depending on the size and the toughness of the seeds available in their environment (Grant and Grant 1999). The ultimate cause of this phenomenon is simply that different “solutions” are optimal for different “problems.” This view is directly associated with the idea that there are trade‐offs to adapt to distinct ecological conditions, for example it is not possible to simultaneously have a small and large beak. Considering that different trait values are optimal in different environments is tantamount to assuming fitness trade‐offs across ecological conditions.

Evolution in heterogeneous environments in the presence of trade‐offs has been extensively studied and can lead to a diversity of outcomes, from coexistence of specialists to evolution of generalist strategies (Kassen 2002; Ravigne et al. 2009). While trade‐offs have been widely documented, they are not always found when investigated in natural populations (reviewed in (Hereford 2009)). Apart from technical issues such as precision of fitness measures, trade‐offs may be undetectable because (i) they are hidden by large variations in fitness caused by unconditionally deleterious or beneficial mutations, (ii) they are weak due to having been attenuated by a long history of adaptation (the environments considered are not outside the range of well tolerated conditions) (Gallet et al. 2014), (iii) environments do not represent different “problems” of nature. Another difficulty is that natural populations were most often exposed to a complex set of ecological conditions in the past, which makes it difficult to link observed adaptive traits with specific environmental conditions. Experimental evolution using microbes in controlled conditions has become essential to address these issues (Kassen 2002; Elena and Lenski 2003; Bell 2008; Garland and Rose 2009; Jansen et al. 2013). With this approach, highly adapted phenotypes can be obtained in response of defined selective pressures, which can be used to reveal fitness trade‐offs among various environments.

Several evolution experiments have measured such trade‐offs across different temperatures (e.g., Bull et al. 2000; Bennett and Lenski 2007), luminosity levels (Reboud and Bell 1997), nutrient sources (Cooper and Lenski 2000; Bataillon et al. 2011), and pH (Hughes et al. 2007; Gallet et al. 2014). Measuring fitness along an environmental gradient, rather than a set of unrelated environments, is of particular interest because it allows for scanning large environmental variation ranges with a quantified “distance” between conditions, estimating whether levels of adaptation can be understood, and making predictions in reference to this “distance.”

Trade‐offs also play a major role in the evolution of antibiotic/pesticide resistance. They are classically measured by evaluating the “fitness cost” of resistance alleles relative to a susceptible allele in the absence of antibiotic (Lenski 1998; Andersson and Levin 1999; Ward et al. 2009; Sousa et al. 2012). From an evolutionary perspective, this cost is a crucial parameter for management strategies (Bonhoeffer et al. 1997; Lenormand and Raymond 1998; Andersson 2006; Hall et al. 2015). This classical view, however, neglects trade‐offs that may occur throughout dose gradients rather than only between treated/nontreated conditions. In fact, it is even often assumed (at least implicitly) that there are no trade‐offs to adapt to different (nonzero) doses of antibiotic or pesticide. With this view, the presence of antibiotics determines a single fitness peak and doses modulate the intensity of selection around that peak. As a consequence, low dose environments (i.e., under the Minimum Inhibitory Concentration, MIC) have been argued to strongly favor the emergence of high dose resistance in natural conditions (Gullberg et al. 2011; Andersson and Hughes 2012). Such low doses could favor the emergence of resistance by providing small fitness advantages to resistance phenotypes that would (i) allow them to persist and remain available upon exposure to a higher dose (reservoir effect) or (ii) allow the occurrence of additional mutations conferring resistance levels that would hardly be achieved in a single step (multiple hit effect). In this view, mutations favorable at low doses confer some advantage at higher doses (so that a multiple hit effect can occur) and high‐dose resistances are also favorable at low doses (so that a reservoir effect occurs). It contrasts with the classical view of evolutionary ecology where trade‐offs are pervasive: different environments correspond to different fitness peaks and select for different phenotypes (e.g., Darwin's finches). Because of these different views, it remains very unclear how adaptation proceeds along dose gradients (Gullberg et al. 2011; Hermsen et al. 2012; Milesi et al. 2016). This issue is critical to understand long‐term adaptation at different antibiotic doses as well as determining the extent to which reservoir and multiple hit effects are important. In this paper, we use experimental evolution to study dose‐specialization of *Escherichia coli* to several doses of an antibiotic, nalidixic acid.

## Methods

### EXPERIMENTAL OVERVIEW

**Figure 1:**
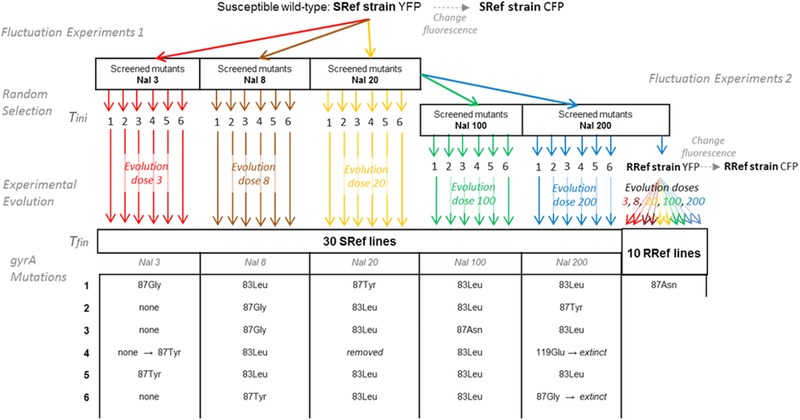
Schematic experimental protocol Schematic experimental protocol. Mutants screen and experimental evolution in different doses of Nal (μg/mL) are represented by the colored arrows. Six experimental lines were evolved in each nalidixic acid (Nal) dose from a random selection of resistant mutants (SRef lines). Two lines additional lines per evolution dose (RRef lines) were evolved from a single resistant mutant screened at Nal200 termed RRef strain and also used as the competitor for fitness assays in presence of Nal. Down, the sequenced resistance mutation in the *gyrA* gene at T_ini_ as well as at T_fin_ (only SRef line 4 in Nal 3 changed between T_ini_ and T_fin_).

We experimentally evolved eight resistant lines of *Escherichia coli* at five doses of nalidixic acid (Nal) (denoted “evolution doses” or “ED”: 3, 8, 20, 100, or 200; all doses are given in μg/mL but these units are not repeated below) for ∼400 generations in order to “push” them close to their optimal phenotype. We started with a susceptible reference strain, or “SRef” strain, corresponding to the 10,000th (abbreviated 10 K) generation of Lenski's long‐term adaptation experiment (*E. coli* strain REL4536). First, we obtained a highly resistant reference strain (“RRef” strain) by screening twice SRef for antibiotic resistance (first screen at Nal 20, second at Nal 200). We then evolved two series of strains at the different Nal doses. In the first main series, six resistant lines per ED (30 lines total) were initiated from independent resistant mutants of SRef obtained after a screen at the same dose than the ED (or a double screen for ED100 and ED200, see below). These lines (hereafter “SRef lines”) provided a diversity of first mutational step and evolved from the screen at a given Nal dose. In the second series, two lines per ED (10 lines total) were initiated from the RRef strain. These lines (hereafter “RRef‐lines”) provided with replicated evolution of the same initial resistant mutant (Fig. 1), but they all started from a screen at high dose (Nal200). In addition to these lines adapting to different Nal doses, we included two types of control lines evolving in absence of antibiotics. First, two control lines (“control10K” lines) were initiated with the SRef strain. SRef is well adapted to the laboratory conditions with no antibiotics, so these controls were useful to determine the extent to which the lines adapted to our lab conditions (rather than to the antibiotics). Second, we initiated four lines with the initial strain of Lenski's experimental evolution (“control0K” lines). This strain is not initially adapted to our lab conditions, so these controls are useful to compare the adaptive trajectory in our conditions with those of Lenski's experiment. SRef and RRef lines were initiated from SRef and RRef strains expressing fluorescent YFP proteins. SRef strain (resp. RRef strain), expressing fluorescent CFP proteins, was used as the reference competitor for fitness assays in absence (resp. presence) of Nal (see below and Fig. 1). We measured the relative fitness of all lines, after the screen (Tini) and after 400 generations of evolution (Tfin) at six different Nal doses (denoted “measure dose” or “MD”: 0, 3, 8, 20, 100, or 150). Bacterial strains.

All lines were derived from the *E. coli* strain REL4536 (SRef), corresponding to the 10,000^th^ generation of Lenski's long‐term adaptation experiment to Davis minimal broth DM25 (Lenski and Travisano 1994). SRef is susceptible to nalidixic acid (Nal) with a Minimum Inhibitory Concentration (MIC) to Nal equal to 2.6. Cyan and yellow fluorescent proteins (CFP, YFP) genes were previously introduced in SRef and REL606 (the ancestral strain of Lenski's LTEE) chromosomic DNA (Gallet et al. 2012).

Six SRef‐YFP resistant mutants were randomly chosen from each of three sets of single mutants, obtained in Harmand et al. 2017 using fluctuation assays at doses 3, 8, and 20, to initiate experimental SRef lines at doses 3, 8, and 20 (Fig. 1). SRef mutants resistant at Nal100 and 200 could not be obtained in one fluctuation assay as the resistance mutation rate was too low at these doses (at dose 20, this rate is already as low as 5.10^−7^). They were obtained via an additional fluctuation assay (protocol as in Harmand et al. 2017) at doses 100 and 200, respectively, starting from 20 independent mutants obtained from the previous screen at dose 20. We refer to them, here and below, as “double” mutants for convenience, as they are very likely to carry two mutations, but we cannot formally exclude that they carry more mutations. Six “double” mutants obtained in this way initiated the evolution lines at doses 100 and 200 (Fig. 1). An additional “double” mutant screened at dose 200, the RRef strain, initiated two evolution lines at each ED (resulting in 10 RRef lines). The YFP gene was replaced by the CFP gene in the RRef initial genotype (see methods in Gallet et al. 2012) to run competition experiments (see after).

### ANTIBIOTIC AND CULTURE MEDIA

Experimental evolution and competitions were performed in DM250 medium, that is Davis minimal broth, as used in the LTEE (composition as in Lenski 1988) but with 10 times more glucose (0.25 g/L). The addition of glucose increases bacterial carrying capacity and allows for more accurate fluorescence measurements, while leading to very similar fitness measures in the LTEE (Cooper et al. 2001; Gallet et al. 2017). We thus assumed that the SRef strain is already well adapted to DM250, so that the antibiotic is the dominant selective pressure during the experiment. This assumption was checked by monitoring fitness variations of the control lines in absence of antibiotics. Fresh medium DM250 supplemented with Nal was prepared weekly and kept protected from light at 4°C. Our first dose of evolution (3) was chosen just above the MIC (2.6), and the different doses used span a wide range of concentrations (up to 80x the MIC). We did not investigate adaptation to doses below the MIC because (i) key issues lie in adaptation to above MIC doses, where resistance matters, and (ii) it is not possible to screen a mutant below the MIC, making the comparison impossible with other (above MIC) doses, since the first step of experimental evolution would necessarily differ.

### EXPERIMENTAL EVOLUTION

The experimental evolution contained the 40 independent resistant lines (30 SRef lines and 10 RRef lines) divided among the five evolution doses and the six control lines (two initiated with the SRef‐CFP strain, two with the REL606‐CFP strain and two with the REL606‐YFP strain) evolving at dose 0. All lines were distributed into four 24‐wells lidded microplates with 1 mL medium per well, with the six Nal concentrations allocated in each plate. Inoculated wells were alternated with noninoculated wells following a checkered pattern, to control for contaminations. The plates were incubated at 37°C, 200 rpm in a water‐saturated atmosphere (sealed box). Every 24 h, 10 μL of the cultured lines were transferred to new wells containing 1 mL of fresh medium and incubated in the same conditions. The protocol was applied for two months corresponding to ∼400 generations of evolution after what the lines were stored as glycerol stocks.

### SEQUENCING

All lines were sequenced at the *gyrA* gene at T_ini_ and T_fin_. This sequence covers most of the promoter and 32% of the *gyrA* gene and its complete quinolone resistance‐determining region (Hopkins et al. 2005). PCR was performed on colonies, using the mix: 10 μL of 2X Phusion Master Mix (ThermoFisher Scientific, Waltham, MA), 1 μL of F‐primer 5′‐AGACAAACGAGTATATCAGGCA [position in *gyrA* sequence: ‐120pb to ‐101pb], 1 μL of R‐primer 5′‐TTTACCAGTTCCGCAATCTTCTC [position in *gyrA* sequence: 823pb to 845pb], 8 μL sterile distilled water; and the PCR program: 5’ 95°C, 35 cycles of [1’ 95°C,1’ 61°C, 2.5’ 72°C] and 5’ 72°C). Sequencing was performed using Eurofins mwg operon (Eurofins, Luxembourg). Mutations in *gyrA* gene were identified by comparing the mutant sequences to that of the SRef strain.

### COMPETITION EXPERIMENTS

Competitions experiments were performed from glycerol stocks at T_ini_ and T_fin_. The “fitness profile” of each line (YFP) across the dose gradient was determined by competitions against the RRef‐CFP strain in DM250 at the five measure doses (3, 8, 20, 100, 150). Ecotoxicological measures such as MIC (minimum inhibitory concentration) are often used to characterize resistance evolution. However, competitive fitness is better suited in our context, to quantify adaptation and the evolution of specialization. For instance, two lineages with similar MIC can have very different competitive abilities, below their common MIC. The measure dose 150 was preferred to dose 200 because the latter exhibited high growth inhibition of the RRef strain, such that fluorescence measurements were close to the detection limit. The fitness costs of resistance were measured from competitions in absence of Nal (dose 0) against the strain SRef‐CFP.

Competition assays were performed in the following procedures. 1:1 volumic ratio competition mixes were prepared from saturated cultures. Fluorescence signals of these mixes were measured on a Tecan Infinite 200 (Tecan, Männedorf, Swizerland) prior to the competition (*t*_0_). Competitions were initiated by inoculating 2 μL of the competition mix into 200 μL of DM250 (and a given dose of Nal). Fluorescence signals were measured again after 24h of growth (*t*_24_) in the same conditions as the evolution experiment (37°C, 250 rpm, water saturated atmosphere). Each competition was repeated at least four times at different dates. We assumed that fitness effects were transitive between genotypes competing in the same dose. Deviation from transitivity as measured in Gallet et al. (2012) in a similar setting, are very small (10^−3^ at most in these experiments).

The selection coefficient per generation associated with each competition was estimated as:

(1) $$s\;=\frac{1}{g}\;\;\left(Log{\left(\frac{n_{\mathrm{YFP}}}{n_{\mathrm{CFP}}}\right)}_{t_{24}}-\;Log{\left(\frac{n_{\mathrm{YFP}}}{n_{\mathrm{CFP}}}\right)}_{t_{0}}\;\right)$$

with $\frac{n_{\mathrm{YFP}}}{n_{\mathrm{CFP}}}$ the ratio of frequencies of YFP and CFP cells estimated from the ratio of YFP on CFP fluorescences in the mix using an experimental calibration curve. The constant g = 6.64 was used to scale the selection coefficients per generation. It approximates the number of divisions, assuming full regrowth from a dilution by 1/100 over a 24 h assay. “Fitness” was denoted as the selection coefficient of a line relative to the RRef strain, and “fitness cost” as the selection coefficient of a line relative to the SRef strain (for measures in the absence of antibiotics).

### STATISTICAL ANALYSIS

Fitness measures were analyzed in a linear‐mixed model (lmer in R 3.2.0, R Core Team) with fixed effects on *lineID*, *MD* (six doses) and *time* (T_ini_ and T_fin_). Random effects included plate and date of the competition assay. The model was used to estimate the fitness values at T_ini_ and T_fin_, the fitness changes between T_ini_ and T_fin_ (pairwise comparisons) of each line in each MD, and their standard errors (performed with mvt adjustments with the lsmeans R package), which are all represented in figures. Polynomial fits of the fitness profiles of evolved lines (from the fitness estimates at T_fin_ of the previous model) in each MD were performed as functions of the log‐ED of lines (nls function of the R Stats package). The fits were done for the SRef lines alone or for both the RRef and the SRef lines for the comparison of the final fitness profiles of lines evolved in the same ED but initiated from mutants screened at different doses.

## Results

### CONTROL LINES

As expected, fitness of control10K lines in MD0 did not vary significantly over time (*P* value = 0.773) nor between replicates (*P* value = 0.270) (Fig. S1). This result confirms that SRef is already well adapted to the experimental conditions and in particular to the medium, which is the same as the DM25 used in Lenski's experiment but with 10 times more glucose. Hence variation in fitness during the experiment results predominantly from adaptations to the presence of Nal. The relative fitness of CFP SRef strain versus YFP SRef strain (cost of the fluorescent markers) was estimated at 0.033 ± 0.024 per generation. In contrast, but as expected, the fitness of control0K lines shows a significant increase through time (*P value* < 10^−4^) due to adaptation to experimental conditions (Fig. 1). Three of the replicates of the control0K lines were mutually not different (*P values* > 0.4). The last replicate differed only slightly from the three others due to a fitness jump at generation 400 (*P value* = 0.067). The global fitness trajectory of the control0K lines is consistent with that expected from the similar 400 generations evolution in DM25 in the LTEE (Lenski and Travisano 1994). These results confirm that the tenfold increase in glucose in DM250 does not cause substantial differences in evolutionary trajectories (see also Cooper et al. 2001; Gallet et al. 2017 on this observation).

### RESISTANCE MUTATIONS IN THE *gyrA* GENE

In *E. coli*, the DNA gyrase is the target of Nal and many of the known resistance mutations occur in the *gyrA* gene coding for the GyrA subunit of this enzyme (Yoshida and Bogaki 1990; Hooper 1999; Harmand et al. 2017). Sequencing revealed that three different *gyrA* mutations (83Leu, 87Tyr, and 87Gly) and some non‐*gyrA* mutations (four lines) were initially sampled across the sets of single resistant mutants screened at the doses 3, 8, and 20 (Fig. 1). Evolution lines at ED100 and 200 were initiated with resistant mutants screened twice, and are most probably double mutants (Fig. 1). In all cases, a maximum of one mutation within the *gyrA* sequence was initially present (83Leu, 87Asn [also present in RRef strain], 87Tyr or 119Glu).

Two SRef‐lines evolving at ED200 (initiated from the *gyrA* mutants 87Gly and 119Glu) went extinct early in the evolution experiment. One of the SRef‐lines evolving at ED20 proved to be initially polymorphic for the *gyrA* resistance mutation. This line was removed from later analysis in order to avoid confusion in the data interpretation. One of the two RRef‐lines evolving at Nal3 was contaminated at one point and removed from the experiment. In all other lines, no additional mutations than the ones detected at T_ini_ were found in the *gyrA* sequence at T_fin_. Among the four lines without *gyrA* mutations initially (hereafter “non‐*gyrA”* lines), only one line acquired a *gyrA* mutation (87Tyr) during the evolution experiment.

### EXPERIMENTAL EVOLUTION OF SPECIALIZATION ACROSS DOSES

**Figure 2:**
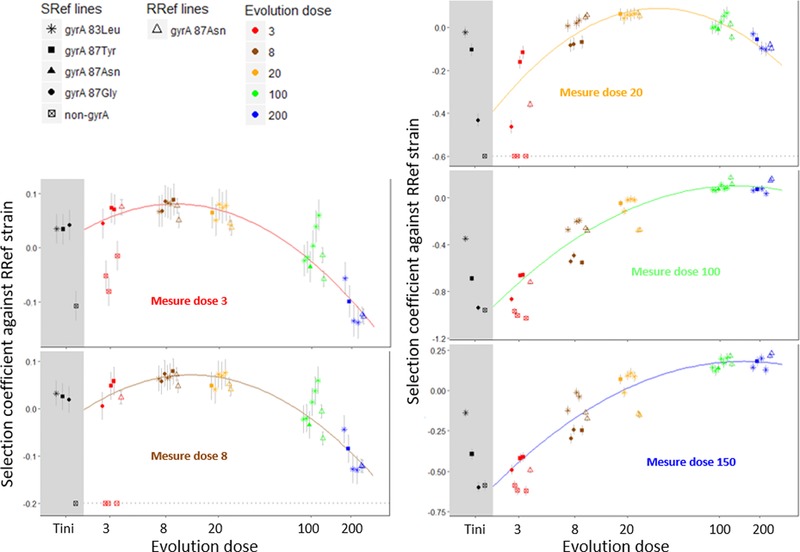
**Fitness profiles of the SRef and RRef lines across evolution doses** after the screen (within the grey area, only mutants screened in one step are shown) and after 400 generations of experimental evolution (within the white area). The *y*‐axis indicates the fitness difference between SRef and RRef lines and the fitness of the RRef strain used as the competitor in all comparisons. Competitions were performed at different measure doses. Each panel corresponds to one measure dose. Mean selection coefficients across replicates are indicated with standard errors. Fitness measures were made for lines evolved at different doses (*x*‐axis). Values of selection coefficients are not comparable across measure doses (because the fitness of the RRef strain varies among measure doses) but fitness ranks are meaningful. For better readability, points are slightly spread on the *x*‐axis at each dose. Each dot corresponds to a different evolved line, with the color corresponding to the evolution dose of that line (see legend) and the symbol to the identity of the initial mutation (different *gyrA* resistance mutation or non‐*gyrA* mutations are indicated in the figure legend). On each panel, a curve of the corresponding measure dose color shows the fitted parabolic relationship between the log‐evolution dose and the selection coefficients of the *gyrA* lines. The dots placed on the horizontal gray dotted lines at MD8 and MD20 where set artificially at this threshold value for the readability of the figure, as they have selection coefficients that were measured to be largely lower than this threshold. Representing their exact value would expand the range of values on *y*‐axis, which would significantly reduce readability for the bulk of the data.

Figure 2 illustrates the fitness of evolved lines (SRef and RRef lines) at generation 400 measured in the different Nal measure doses. This fitness is evaluated against the same reference competitor (RRef strain) for all lines. However, because fitness of this RRef strain varies across different antibiotic doses, comparison among lines are only straightforward within a given environment (i.e., at a given measure dose). However, measures across environments are are comparable to within a constant, so that the shape or location of fitness profiles and fitness ranks can be compared across measure doses (i.e., across panels of Fig. 2). The first striking observation is that, except for the three lines that did not have a *gyrA* mutation at generation 400 (red empty squares, more on these below), all fitness profiles show a regular quadratic pattern with log‐ED. Fitted curves are shown on each panel and Table 1 provides corresponding estimates and their standard error. These profiles demonstrate a strong pattern of specialization explaining c.a. 80% of the fitness variation. At MD8, MD20, and MD100 the profile maximum falls close to the corresponding ED (see also Table 1 SRef lines fit). It shows that the final fitness of a line is largest when the difference between the measure dose and its evolution dose is smallest. Lines best adapted to a dose were those that evolved at that dose, the second best adapted were those that evolved at a slightly different dose etc. This general pattern is also observed but less strong at MD3 and MD150, where the maximum is estimated at a dose of 11 and 99, respectively (see Table 1 SRef lines fit). The second important observation is that the fitness at generation 400 still depends on the first mutational steps. In particular, the three SRef lines (out of four initially) that do not have a *gyrA* mutation show a large fitness lag compared to all other lines, at all MD. Given this large and consistent difference, these lines were not included in the fit of the average fitness profiles among ED represented on Figure 2. Interestingly, these lines did not acquire *gyrA* mutations after the initial screen, despite that these mutations are frequent and provide a strong fitness benefit. The effect of the first mutational step is also visible on *gyrA* lines, but is very small. In several cases, lines with the same initial *gyrA* mutation tend to group together for a given ED and MD (see e.g., ED8 lines with 83Leu mutation at MD 20, 100, and 150). Similarly, RRef lines (which all started from the same highly resistant mutant), tend to group together as well. These effects remain, however, minor compared to the overall trend. Even the RRef lines, which had a quite different starting point than most lines, show a final pattern very consistent with the general trend of SRef lines (Fig. 2 and Table 1 fit with or without RRef Lines).

**Table 1:** Fit of fitness profiles of the *gyrA* mutated evolved lines SRef alone (left) or SRef and RRef lines grouped together (right) as a second‐order polynomial of their log evolution dose (ED)

	*gyrA* SRef lines				*gyrA* SRef and RRef lines			
Measure dose (Log‐measure dose)	Fitted curvature	Fitted optimum in Log‐Evolution Dose	Optimal Evolution Dose estimate	% Variance explained	Fitted curvature	Fitted optimum in Log‐Evolution Dose	Optimal Evolution Dose estimate	% Variance explained
**3** (1.10)	0.020 ± 0.004	2.37 ± 0.20	11	84.9%	0.019 ± 0.003	2.23 ± 0.20	9	85.9%
**8** (2.08)	0.021 ± 0.004	2.56 ± 0.17	13	80.4%	0.021 ± 0.003	2.52 ± 0.14	12	83.1%
**20** (3.00)	0.052 ± 0.009	3.58 ± 0.10	36	68.1%	0.057 ± 0.007	3.54 ± 0.07	34	71.2%
**100** (4.61)	0.070 ± 0.013	4.57 ± 0.25	96	90.1%	0.057 ± 0.011	4.90 ± 0.32	134	89.3%
**150** (5.01)	0.051 ± 0.009	4.59 ± 0.24	99	91.1%	0.041 ± 0.008	5.02 ± 0.36	152	89.3%

### THE EMERGENCE OF DOSE SPECIALIZATION

**Figure 3:**
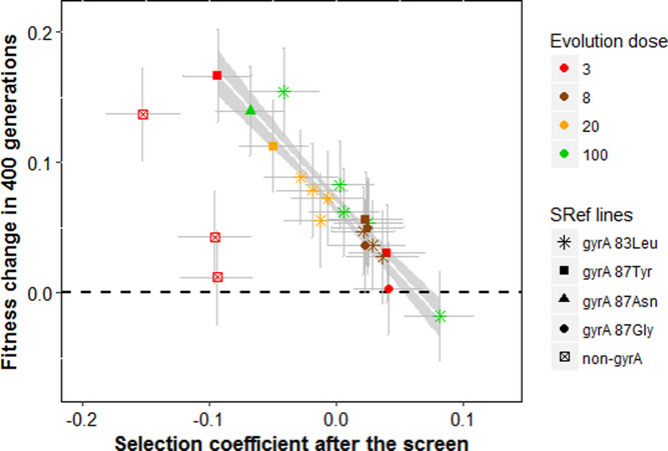
**Fitness change per resistant line after 400 generations of evolution versus initial fitness in the measure dose corresponding to the evolution dose** (lines evolved at dose 200 were measured in dose 150, so were not included here). Symbols indicate the *gyrA* mutations sequenced in each line while colors indicate the dose at which they were selected and evolved. The best model yields different intercepts for non‐*gyrA* lines (open squares) and *gyrA* lines (all other symbols, the corresponding model indicated by the gray zone). Selection coefficients after the screen (*x*‐axis) correspond to the fitness differences with the RRef strain used as the competitor in each case. Fitness change (*y*‐axis) refers to the difference of selection coefficients against the RRef strain after evolution and after the screen for the same line. Dots and error bars are mean values across replicates and the corrected standard errors associated with these values.

Studying a large sample of mutants screened at different doses in the same system showed that little dose specialization occurred after a single mutational step (Harmand et al. 2017). In agreement with this previous study, the fitness profiles of the resistant mutants SRef across screen and measure doses do not show a pattern of dose‐specialization here (the fitness of the SRef resistant mutants obtained from a single screen are shown in Fig. 2, but see Harmand et al. 2017 for a comprehensive dataset). Most of the dose‐specialization occurred after acquisition of the first resistance mutation. Figure 3 demonstrates the change in fitness of each SRef line between the resistance screen (T_ini_) and generation 400 of evolution (T_fin_) in the MD corresponding to its ED. SRef lines evolved at ED200 are not shown as their fitness were not measured at MD200 (see Methods). For all lines, the fitness change was positive or not significantly different from zero at the dose in which they evolved. Among gyrA lines, the trend shows striking regularity independently of the evolution dose and of the identity of the gyrA mutation carried initially (Fig. 3). Over all these lines, in their respective ED, the fitness change is negatively and linearly related to the initial fitness (Fig. 3, *P* value < 10–4), with a slope close to –1. The trend is also observed among non‐gyrA lines but with a different intercept (*P*‐value = 0.002) and similar slope of about –1. This pattern is expected when evolution corresponds to convergence to a fitness peak, as it indicates that the fitness changes entirely compensate initial maladaptation. This pattern excludes a strong role for “chance” or transient “historical” effects (Travisano et al. 1995). This “rule of declining adaptability” has been shown to hold empirically and theoretically in some models of adaptation, notably involving a single peak fitness landscape (MacLean et al. 2010; Perfeito et al. 2014; Couce and Tenaillon 2015; Martin and Roques 2016), and while linearity is expected at all time, a slope equal to –1 indicates that populations reached mutation–selection balance around that peak (Martin and Roques 2016). Figure 2 shows the fitness variation in each MD for all ED: this linear trend tends to be conserved across all MD, but with an intercept that decreases when the difference between the ED and the MD increases. The observation that this pattern was similar across all evolution doses is therefore consistent with the interpretation that all populations approached closely their phenotypic optimum. This conclusion is further corroborated by the observation that both SSref and RRef strains converged to the same pattern across ED and MD, despite starting from quite different starting points (Fig. 2). On average, the lines that evolved at low ED (ED3, ED8, and ED20) have a low fitness at T_ini_ at high doses (ED100 and ED150) and show an adaptation (or no change) in all MD, indicating that evolving at low dose increases fitness at high dose. A different pattern is observed for lines evolved at high doses. While they adapted to their ED, they consistently and regularly “deadapted” to low doses.

### FITNESS TRADE‐OFFS ACROSS DOSES

**Figure 4:**
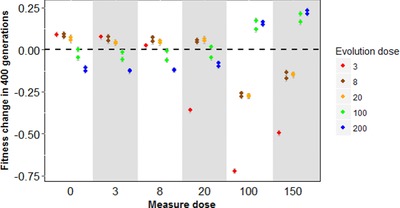
Fitness change of the RRef‐lines compared with their ancestor (the RRef strain) Fitness change of the RRef‐lines compared with their ancestor (the RRef strain). The RRef lines evolved during 400 generations in different Nal antibiotic in μg/mL doses (colors) at different measure doses (*x*‐axis). Each dot represents the mean value for a line across replicates associated with its standard error. Positive values indicate an adaptation to the measure dose whereas negative values stand for a counter‐adaptation.

The emergence of specialization is usually caused by the occurrence of fitness trade‐offs across environments. With SRef lines, it is not straightforward to observe these trade‐offs since the lines start with different fitness after the initial screen. In other words, some are closer from their optimum than others. A convenient way to have a clearer view of these trade‐offs from the start is to look more closely at the RRef lines. These lines all start from the same genotype. Hence it is easier to see how adapting to one dose impact fitness in other environments. The change in fitness of the RRef lines over the course of the experiment is shown on Figure 4. Here again, the fitness changes follow a regular pattern according to the dose of antibiotic. Evolution at a given dose leads to an increase in fitness at that dose, but to a decrease in fitness in environments that are largely different from that ED. For instance, the fitness of lines that adapted to high ED decreased at low dose (and vice versa). These results suggest that there are strong fitness trade‐offs on the phenotypic traits related to the resistance at low, intermediate and high Nal doses. This fitness variation is most probably caused by the spread of beneficial mutations that have deleterious effects in doses very different from the ED. Some of these effects might be caused by deleterious mutations hitchhiking along with these beneficial mutations, as can occur with asexuality. However, in both case it requires that the effects of mutations vary gradually among doses to lead to the regular pattern of specialization. Fluoroquinolone antibiotics are known to be mutagenic (Cook et al. 1966), at least in susceptible strains. However, this possible mutagenicity cannot explain our results. Specialization occurs similarly at all doses, and loss of fitness in doses very different from the ED is not specific to strains adapting at high ED (see e.g., ED 3, 8, 20 on Fig. 4).

### EVOLUTION OF THE COSTS OF RESISTANCE

**Figure 5:**
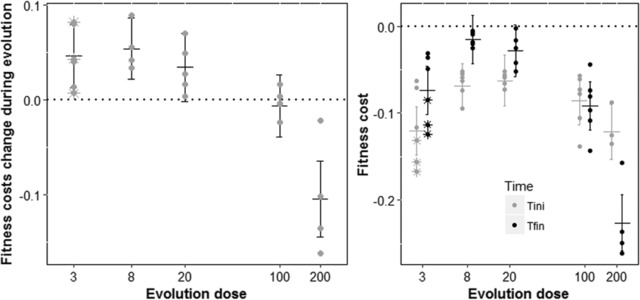
Evolution of the costs of resistance across Nal doses Evolution of the costs of resistance across Nal doses. Bars represent means ± SD among lines evolved at the same dose. Dots are mean values of fitness change (left panel) or selection coefficient (right panel) for each different *gyrA* line while stars indicate the non‐*gyrA* lines. On the right panel, mean selection coefficients are plotted after the screen (in grey) and after the 400 generations of evolution in antibiotic (in black) in the corresponding evolution dose. On the left panel, we see that the fitness costs are reduced when evolving at low doses, but that they increase when evolving at high dose. On the right, we see that fitness costs are globally larger at high evolution dose, but also that non‐gyrA mutant at the lowest dose exhibit a very strong cost.

At T_ini_, all resistant mutants were costly (Fig. 5, right panel). Those initial costs were similar among the different *gyrA* resistance mutations of single‐mutants (ED3, ED8, ED20) but higher for non‐*gyrA* mutations. They were also variable between single‐mutants and “double”‐mutants of ED100 and ED200, even when they carried the same *gyrA* mutation. The change in cost between T_ini_ and T_fin_ is negatively correlated with the ED (*P*‐value < 10^−6^, left panel of Fig. 5 and MD0 on Fig. 4). Adaptation to high ED led to an increase in the fitness cost of resistance. On the contrary, adaptation to lower ED (ED3, ED8, ED20) eventually led to smaller costs. At T_fin_, the *gyrA* lines are increasingly costly with higher ED (filled black circles on Figure 5 and Fig. 3 for specification of the *gyrA* mutations). The non‐*gyrA* lines (present only at ED3) have generally higher costs than the *gyrA* lines (see also Harmand et al. 2017), which leads to a higher mean cost at ED3 than ED8 or 20, when all lines are pooled.

## Discussion

In order to investigate the existence of fitness trade‐offs along dose gradients, we experimentally evolved resistant lines of *E. coli* at different antibiotic doses. Our results showed that lines diverged after 400 generations and rapidly specialized to their evolution dose. This pattern of specialization was highly regular across doses and achieved convergently by lines with different degree of maladaptation. It was achieved symmetrically for different starting points. In particular, highly resistant lines specialized at lower dose, while experiencing a fitness decrease at high dose. These patterns rule out the possibility that this pattern of dose‐specialization resulted from a transient dynamics. They reveal the existence of pervasive fitness trade‐offs in adaptation to different doses of an antibiotic gradient.

### FITNESS LANDSCAPE ALONG A GRADIENT OF ANTIBIOTIC DOSES

The occurrence of fitness trade‐offs strongly supports the idea that doses can be considered as different “environmental conditions” or different selective constraints in terms of adaptation. Using the well‐known representative metaphor of adaptive fitness landscape, this means that the phenotypic position of the fitness peak is changing along the dose‐gradient. The fitness profiles of our evolved lines also appear to be remarkably regular along the dose gradient, suggesting that it should be possible to make predictions for other, unused ED. The optimal MD very consistently tracks the ED and fitness is monotonously decreasing from the optimum as shown by quadratic relationships with log‐dose of antibiotic (Fig. 2). These results provide support in modeling adaptation along ecological gradients using a gradual shift in phenotypic optimum, as often proposed theoretically (e.g., Lynch and Lande 1993; Kirkpatrick and Barton 1997).

In a previous study (Harmand et al. 2017), we investigated mutational and selective patterns of resistance from single resistant mutants across the same antibiotic dose gradient. These mutational patterns did not allow determination of whether different doses corresponded to different phenotypic optima, selection intensities around the same optimum, or both. Here, we show that phenotypic optima vary among doses. This finding does not exclude variations in the selection intensity across doses, but such variation alone, without a variation in the position of the peak, would not account for our data.

Finally, the net adaptive fitness change during our experiment was predictable across the different lines: it scaled linearly with initial maladaptation (Fig. 2). This pattern is known as the “rule of declining adaptability” and is predicted by simple fitness landscape models (Couce and Tenaillon 2015; Martin and Roques 2016). Furthermore, the slope of this trend was close to –1, consistent with the view that the different lines reached a position close to their respective dose‐specific optimum. Overall, this study suggests that evolution across antibiotic dose gradients conforms closely to peak shift models. Further experiments, with different antibiotics, and longer time scales, will be very interesting to check the generality and robustness of this conclusion.

### CONSEQUENCES FOR THE EVOLUTION OF RESISTANCE

From a practical point of view, the existence of different phenotypic optima regularly arranged along a dose‐gradient has several implications. Selection at low and intermediate doses can indeed promote adaptive steps toward high resistance (consistent with a reservoir effect). Furthermore, phenotypes evolved at intermediate doses are better adapted to high doses than phenotypes evolved at low doses (consistent with a multiple hit effect). Additionally, long‐term selection at low doses will not result in high fitness (meaning optimal phenotypes) at high doses, and as a result, long‐term selection at high doses will not select for high‐fitness phenotypes at low doses. These conclusions have important implications for understanding and modeling the evolution of resistance in the field, under heterogeneous conditions.

To our knowledge, no previous study has clearly established the pattern of dose‐specialization along a gradient so far. First, many studies have investigated the fitness of resistant mutants from field samples (clinical isolates in the case of antibiotic resistance, several examples reported in Davies and Davies 2010). However, except in few cases (e.g., Labbe et al. 2005; Milesi et al. 2016), the ecological context (and dose) in which those mutations were selected for is usually unknown and potentially complex. Simply knowing the fitness of a mutation in the environment where it was selected for is obviously insufficient to conclude about trade‐offs. The absence of well‐controlled conditions in the field makes field samples difficult to interpret. Second, some experimental studies looked at short‐term antibiotic resistance evolution by screening mutants under controlled conditions (Thulin et al. 2015; Harmand et al. 2017). In general, this short‐term evolution cannot easily reveal trade‐offs across environmental conditions, especially when the initial type is highly maladapted (Bataillon et al. 2011; Martin and Lenormand 2015), as it is the case for resistance when starting from a susceptible wild‐type. Last, studies that used long‐term evolution of resistance in laboratory conditions often focused on the evolution of the cost of resistance, that is the trade‐off between absence versus a given dose of antibiotic (reviewed in Melnyk et al. 2015) and/or did not assay directly fitness across doses (e.g., Gullberg et al. 2011; Hughes and Andersson 2012). While it is clear that such studies provide key insight for resistance management, studying fitness variations across full gradients and in particular at low doses is probably critical to understand long‐term resistance evolution in natura and to develop accurate management models.

Due to the occurrence of shifted optima across doses, the long‐term evolution of the cost of resistance largely differs across evolution doses (Fig. 5). Previous studies have highlighted that fitness costs are highly variable (e.g., depending on mechanisms) and difficult to predict in a general way (Melnyk et al. 2015; Vogwill and Maclean 2015). Our results show that taking into account different dose‐environments is a key element (see also Westhoff et al. 2017). Because low dose optima are closer to dose zero than high dose optima, the dynamics of cost evolution can be radically different at a given dose. We found a negative correlation between the fitness cost changes and the evolution dose (Fig. 5 and ED0 in Fig. 4). At low doses, initial costs are quickly compensated, suggesting that the first mutational step overshot the phenotypic optimum at low ED. After compensation, they become very small, as expected since selection intensity has to be much lower around the ED0 optimum than around optima with antibiotics. At higher doses, however, costs increase through time. The presence of different optima across doses explains this pattern and can thus provide a powerful conceptual framework to understand the large variability of fitness costs observed previously in long‐term studies.

### HISTORICAL CONTINGENCY OF ADAPTIVE TRAJECTORIES

“Historical contingency” or “mutation‐order” effects have been widely discussed in the literature to describe the dependence of adaptive trajectories on initial conditions (Elena and Lenski 2003; Lenormand et al. 2009, 2016; Lobkovsky and Koonin 2012). Our results show that the pattern of specialization is general among *gyrA* lines but still dependent on the identity of the first *gyrA* mutation after 400 generations of evolution. However, the good alignment of the initial fitness versus the fitness change seems to indicate that those lines progress toward the same optimum within an ED. It is thus possible that the lines reach closer to the optimum of their ED but still progress toward this optimum after the 400 generations of evolution. Alternatively, the non‐*gyrA* lines seem to progress toward a different point than the *gyrA* lines (there is a large and significant “mutational module” effect on the regression of the initial fitness versus the fitness change). After 400 generations of evolution at ED3, their fitness profiles are still largely different to those of the *gyrA* lines indicating a strong historical contingency between those “mutational modules” of resistance. Contrary to the specialization pattern, this divergence in fitness patterns between the *gyrA* and the non‐*gyrA* lines was already observed immediately after the first resistance screen and interpreted as the occurrence of selective covariances across traits in Harmand et al. 2017. Together, the results of these two studies put forward the *gyrA* and non‐*gyrA* resistant lines as good candidates to investigate the effects of historical contingency and selective covariances on long‐term adaptive trajectories.

Studies of resistance evolution (very broadly defined, that is resistance to antibiotics, chemotherapy, insecticides, acaricides, fungicides, herbicides etc) often overlook the possibility that dose gradients may represent different phenotypic challenges, in addition to also representing different selection intensities. Our results show that this simplification is not warranted. They show that the evolution of resistance along antibiotic dose gradients is consistent with classic evolutionary models of adaptation on ecological gradients, where each environment corresponds to one fitness peak. The observed patterns of adaptation and maladaptation are also fully consistent with the occurrence of dose‐dependent optima and show pervasive trade‐offs across doses. Hence, our findings call for more realistic models of resistance evolution in heterogeneous dose conditions that include dose‐specialization. Such models are necessary to better evaluate the impact of low doses of antibiotics that are today ecologically widespread.

## CONFLICT OF INTEREST

The authors declare no conflict of interest.

Associate Editor: K. Lythgoe

## Supporting information


**Fig. S1**. Selection coefficients of control lines evolved in the absence of antibiotic in competition against the non‐evolved wild‐type 10K‐YFP (or 10K‐CFP in the case of 0K‐YFP lines).

**Fig. S2**. **Fitness change of the SRef lines during 400 generations of evolution in different evolution doses (colors) versus their fitness just after the screen of resistance in the different measure dose**s. Symbols indicate the *gyrA* mutations sequenced after evolution in each line while colors indicate the dose at which they were screened and evolved. Error bars represent standard errors of the mean and the fitness change estimated in the statistical model.

**Figure S2b**

**Figure S2c**

**Figure S2d**

**Figure S2e**

**Fig. S3**. **Costs of resistance of the SRel lines evolved for 400 generations at five evolution doses of antibiotic**. Symbols indicate the mutation detected in the *gyrA* sequence or the absence of mutation in the *gyrA* sequence while colors indicate the evolution dose. The dotted horizontal line at 0 corresponds to an equal fitness with the susceptible ancestor 10K, while negative values correspond to a lower fitness of resistant lines. Error bars represent standard errors among replicates.
