# Supplementary figures and images for: Evolution of bacteria specialization along an antibiotic dose gradient
Source: Evol Lett. 2018 May 8;2(3):221–32. doi: 10.1002/evl3.52 (PMC6121860; doi:10.1002/evl3.52)

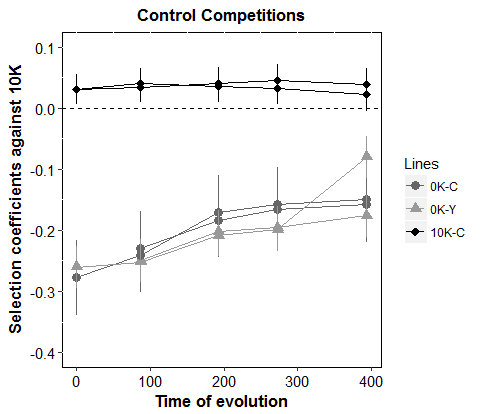

Supplement: Supplementary file 1 — Fig. S1. Selection coefficients of control lines evolved in the absence of antibiotic in competition against the non‐evolved wild‐type 10K‐YFP (or 10K‐CFP in the case of 0K‐YFP lines). [file EVL3-2-221-s001.tif]

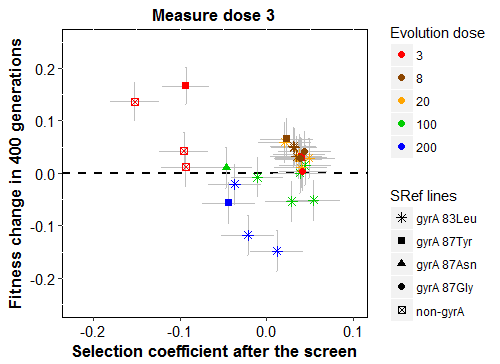

Supplement: Supplementary file 2 — Fig. S2. Fitness change of the SRef lines during 400 generations of evolution in different evolution doses (colors) versus their fitness just after the screen of resistance in the different measure doses. Symbols indicate the gyrA mutations sequenced after evolution in each line while colors indicate the dose at which they were screened and evolved. Error bars represent standard errors of the mean and the fitness change estimated in the statistical model. [file EVL3-2-221-s002.tif]

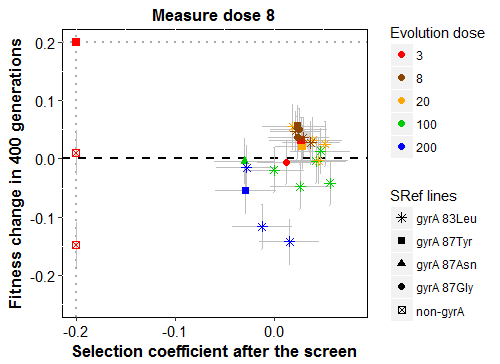

Supplement: Supplementary file 3 — Figure S2b [file EVL3-2-221-s003.tif]

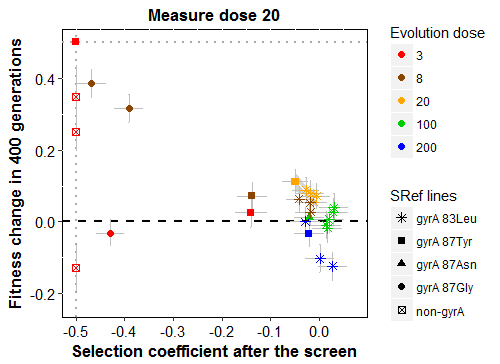

Supplement: Supplementary file 4 — Figure S2c [file EVL3-2-221-s004.tif]

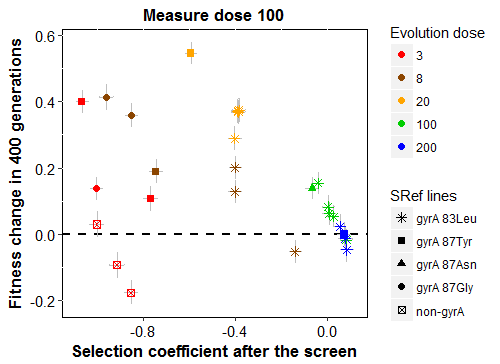

Supplement: Supplementary file 5 — Figure S2d [file EVL3-2-221-s005.tif]

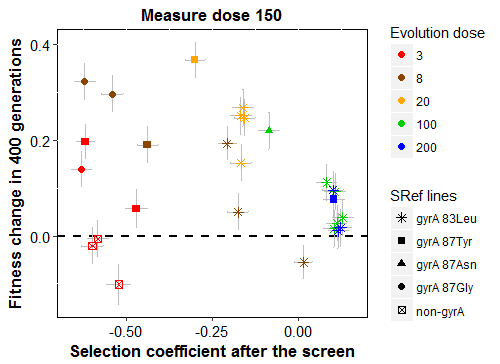

Supplement: Supplementary file 6 — Figure S2e [file EVL3-2-221-s006.tif]

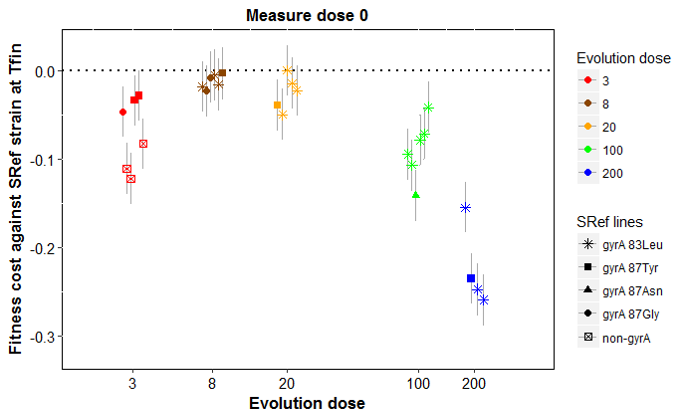

Supplement: Supplementary file 7 — Fig. S3. Costs of resistance of the SRel lines evolved for 400 generations at five evolution doses of antibiotic. Symbols indicate the mutation detected in the gyrA sequence or the absence of mutation in the gyrA sequence while colors indicate the evolution dose. The dotted horizontal line at 0 corresponds to an equal fitness with the susceptible ancestor 10K, while negative values correspond to a lower fitness of resistant lines. Error bars represent standard errors among replicates. [file EVL3-2-221-s007.tif]
